# Cardiac rehabilitation in ACHD: Further investment is now due

**DOI:** 10.1016/j.ijcchd.2024.100564

**Published:** 2024-12-31

**Authors:** Ioannis Kasouridis, Heather Probert, Michael A. Gatzoulis

**Affiliations:** a Adult Congenital Heart Centre and National Centre for Pulmonary Hypertension, Royal Brompton & Harefield Hospitals, London, UK; b Cardiovascular Prevention & Rehabilitation Department, Royal Brompton & Harefield Hospitals, London, UK

## Abstract

It is paramount that we as CHD physicians and health care providers apply an individualised patient approach to assessing and encouraging an active lifestyle to all CHD patients and referring freely patients undergoing percutaneous / surgical interventions orafter decompensated heart failure admissions for cardiac rehabilitation.

Cardiac rehabilitation is an important multidisciplinary intervention that encompasses the full spectrum of exercise prescription, dietary education and psychosocial support.

The evidence for the use of prevention and rehabilitation programmes after atherosclerotic cardiovascular disease events (ASCVD) or revascularisation have been shown to reduce cardiovascular mortality, hospitalizations and myocardial infarctions with a 1A recommendation from the European society of Cardiology (ESC) [1]. Separately in patients with chronic heart failure with reduced ejection fraction (HFrEF), exercise programmes and exercise prescription may improve all-cause mortality, improve exercise capacity and quality of life and reduce hospitalisations (1A level of evidence) and consideration should be given to supervised exercise rehabilitation programmes in patients who can participate (2a-C level of evidence). In addition, exercise rehabilitation has also been shown to be a cornerstone for patients with heart failure with preserved ejection fraction (HFpEF). The ESC sports cardiology section recommends programmes ranging from 12 to 24-weeks in length as providing maximal benefit in exercise capacity and quality of life, encompassing moderate endurance and dynamic resistance training in addition to lifestyle modifications and risk factors (1C level of evidence). By 2028, the goal is for 33 % of all heart failure to be referred to cardiac rehabilitation [2,3].

While the evidence from randomized control trials (RCT) and guideline recommendations in the cohorts mentioned above abound, the same is not the case for the smaller cohorts of patients with congenital heart disease (CHD). A Cochrane review in 2020 focusing on exercise interventions in adults and children with CHD(15 trials including 924 patients) wasn't able to accurately determine the effect of cardiac rehabilitation. A small improvement in peak oxygen uptake (VO_2_, mean difference 1.89 ml/kg/min) and muscle strength (mean difference 17.1N/m) was noticed with cardiac rehabilitation [4]. Given the absence of as yet long-term outcome data on exercise prescription and cardiac rehabilitation in CHD patients, surrogates such as maximal oxygen consumption (VO_2_ max), among other variables, have been used in the literature with reduced VO_2_ values noted depending on the complexity of CHD [5,6].

A prospective study by [7] comprised of 163 patients aged 5–10 years of age with CHD diagnosis showed the participants in the intervention group improving in variables such as exercise duration, maximum power output and daily activity by 11 %. Patients in their intervention group were divided based on their CHD diagnosis severity into 3 groups (acyanotic, cyanotic, cyanotic palliated). Of those, the cyanotic palliated group demonstrated the biggest cardiorespiratory impairment at rest and with exercise. **(**7) In another multicentre study by Duppen in 2015, 93 patients with tetralogy of Fallot or a Fontan circulation, aged 10–25, were included to assess the effect of aerobic exercise intervention over 12 weeks. The exercise group showed a 5 % increase in peak VO_2_ and significant increase in workload measured by metabolic equivalent of task (METs). The study's exercise protocol involved aerobic training three times a week, consisting of 1 h exercising at sub-maximal levels (10 min warm up intervals, 40 min cycling exercise, 10 min period of cool down exercise). There were no reported training related adverse effects and this study further supports the safety and importance of exercise prescription and rehabilitation in CHD patients [8].

Most recently, Amedro et al. conducted a multicentre randomised control trial (QUALIREHAB) enrolling 142 patients with CHD(age range 13–25yrs old, VO_2_ Max <80 %) and implementing a 12 week hybrid cardiac rehabilitation programme. The study looked at the effect of rehab in health related quality of life (HRQoL) as primary outcome and change in cardiovascular parameters and fitness as secondary outcomes. They were able to show that patients following an early hybrid cardiac rehabilitation programme (home and centre based) had a significant improvement in HRQoL) as well as body mass index and increase in physical activity and understanding of their pathology [9].

While it is important to support and encourage all our CHD patients to engage in exercise and referring appropriately for cardiac rehabilitation, it is equally important to make some distinctions among them, such as in patients with cyanotic heart disease and Fontan circulation who need to be closely supervised. A bespoke exercise protocol for each patient needs to be prescribed depending on their needs, condition and abilities. An exercise protocol that favours aerobic versus resistance training is recommended as aerobic exercise limits the elevation of systemic vascular resistance and end-diastolic pressure which can impact exercise tolerance by reducing the stroke volume. In addition, in cyanotic patients’ oxygen supplementation can lead to better exercise performance and increase the duration of the activity at the beginning stages with the goal to withdraw this gradually. Functional capacity at baseline and at the end of an exercise rehabilitation with an objective test such as a CPET is recommended [10].

In particular in the cohort of patients with Fontan circulation, evidence from studies evaluating exercise capacity in high functioning Fontan patients (‘super Fontan’ -defined as max predicted VO_2_ greater than 80 %) have been strongly in favour of regular participation of sports and regular physical activity. Furthermore, resistance training has been shown to increase exercise capacity and prevent skeletal muscle wasting in this cohort. This is particularly important as increased lower limb muscle mass has been associated with an improved ability to augment systemic output during exercise and with increasing cardiac output and filling at rest and exercise. In patients with a Fontan circulation, this could be especially relevant as more blood is pumped back into the venous system with each movement with associated improved in oxygen pulse on CPET data and overall better performance. Lastly, resistance/strength training should be tailored to each patient with advice to avoid straining, including the Valsava manoeuvre with part of the exercise prescription focusing on the development of lower limb musculature to augment the skeletal muscle pump [11].

Prior to planning for cardiac rehabilitation, it is important for functional capacity testing to take place to guide planning and evaluate the functional aerobic capacity as well as risk stratify the patient prior to exercise prescription. If the patient is unable to perform a standard exercise test (using a cycloergometer or treadmill) then an alternative functional test like a 6-min walk test (6MWT) or an incremental shuttle walk test (ISWT) should be performed as a minimum standard and to allow for comparative objective data pre and post the rehabilitation programme. These patients are used to historically poor exercise tolerances so may well under report subjective measures of exercise intensity which can be challenging [12,13].

The European association of preventative cardiology (EAPC) have issued general recommendations for physical activity counselling and prescription with regards to cardiac rehabilitation, however each programme has to be tailored to each patient following appropriate assessment. They recommend submaximal endurance training with gradual increase, adjusting the prescription based on the data from exercise testing as mentioned above. A minimum of 2.5 h/week of moderate aerobic activity, with multiple bouts of more than 10 min, 4–5 days/week. Resistance training is also recommended at least twice a week [14].

## Exercise prescription and rehabilitation

While exercise prescription plays an important role in cardiac rehabilitation, it is beyond the scope of this paper to provide a complete guide as to how to perform this on an individual patient basis. The prescription of physical exercise depends on multiple factors given the complexity and heterogeneity among CHD patients. Different kinds of exercise can have a different effect on the heart with dynamic exercise causing volume loading and static exercise pressure loading.

CPET testing is important to guide the training intensity of exercise prescription by providing data including maximal heart rate (MHR, heart rate reserve, maximal/peak oxygen consumption (peak VO_2_), first ventilatory anaerobic threshold(VAT) and maximal workload. In addition, the use of the Borg scale (Table 1, rate of perceived exertion-RPE) along with a fixed percentage of MHR has been shown to be a useful tool to monitor patients during exercise training. However, in CHD patients where use of a fixed percentage of MHR may not be applicable, the maximal workload or VAT could be used to guide exercise prescription.

**Table 1:** Rate of perceived exertion (Borg scale) and its relationship with maximal heart rate (MHR) achieved during CPET [14].

Borg scale (Range 6–20)	Description of Exercise intensity	Way it feels to patient	% of MHR
6–9	Very light	No muscle fatigue, breathlessness	<35
10–11	Light	Light exercise, easy to talk.	35–54
12–13	Moderate	Fairly strenuous, not so easy to talk.	55–69
14–16	Hard	Heavy and strenuous, as when running or walking fast.	70–89
17–19	Very hard	Very strenuous, very tired and breathless, very difficult to talk	≧90
20	Maximal	Maximum heaviness, unable to talk, can't breathe anymore	100

A 6-step approach model has been proposed from a European group of experts in CHD, sports cardiology and exercise physiologist that included a) medical and surgical history and examination, b) assessment of ventricular function/ventricular volume or pressure loading, pulmonary artery pressure, aorta, arrhythmia, and saturations at rest and exercise, c) recommendation for static component of exercise, d) CPET testing, e) recommendation for.

**Fig. 1:**
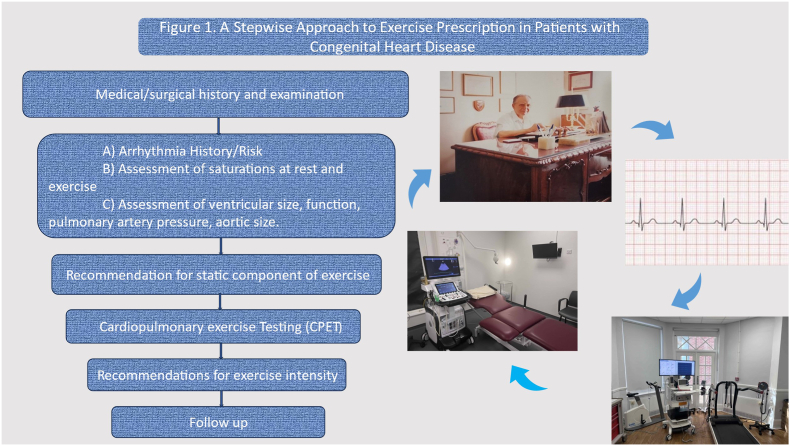
A suggested stepwise aproach to exercise prescription in patients with CHD.

Intensity and follow-up (central illustration-Fig. 1) [6,14].

The specific type of exercise prescription and particularly the level of static exercise should be decided on a case-by-case assessment of the above parameters.

The patient should be counselled to check and monitor their maximum heart rate and any symptoms with the Borg Scale so they can pace themselves during those activities [15,16].

**Fig. 2:**
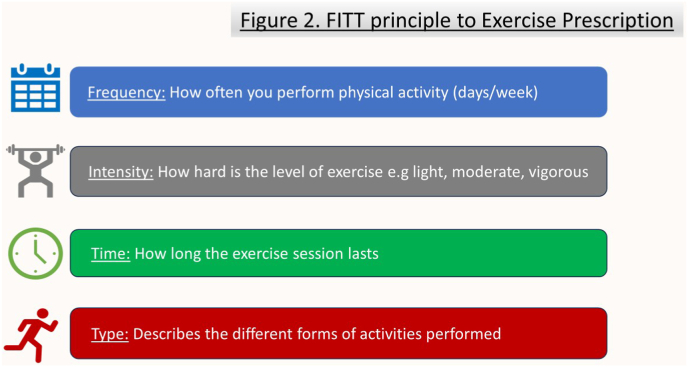
FITT principle to Exercise Prescription.

While more adult based studies are being conducted on the effects of physical exercise and rehabilitation on patients with different types of CHD, a report from Takken et al. from the European Association of Cardiovascular prevention and rehabilitation on paediatric patients can help us extrapolate for our adult population. They support endurance training as the best way to improve individual patient cardiovascular status with any benefit derived from it relating to the ‘dose’ of exercise prescribed and conducted. The use of FITT (frequency, intensity, time and type of activity, Fig. 2) is a useful way for a clinician to adopt in prescribing physical activity to aid cardiovascular fitness and rehabilitation.

It is paramount to emphasize herewith the importance of exercise as a lifelong pursuit not just to our adult patients with congenital heart disease but from the paediatric age, so as to promote a healthy lifestyle and improve health outcomes. Overrestriction based on misperceptions of the risks of exercise in children has been a reason for the lack of engagement early on in life before they make into adulthood, a practice of the past that must be abolished. In the same line, in a study conducted on young adults, only a third requested information on types of safe or appropriate levels of exercise, assuming that all types of exercise were allowed for their condition. Therefore, it is important that each consultation whether in a rehabilitation setting, a pre-op setting or a regular routine outpatient setting covers advice and suitability of exercise as part of the review [9,17,18].

In conclusion, despite robust evidence and guideline recommendations, access and uptake of cardiac rehabilitation and exercise prescription remains globally poor. There is now an ever-increasing body of evidence that physical activity can be safe and a useful adjunct to medical and surgical interventions for CHD patients with appropriate screening and supervision being vital to its success [18].

The advent of home-based, virtual, technology-based models of cardiac rehabilitation, in part expedited by the recent coronavirus pandemic in 2020, should facilitate better access and delivery for patients and promote studies and trials on such an important subject [19].

## CRediT authorship contribution statement

**Ioannis Kasouridis:** Writing – original draft, Writing – review & editing. **Heather Probert:** Writing – review & editing. **Michael A. Gatzoulis:** Writing – review & editing.

## Declaration of competing interest

The authors declare the following financial interests/personal relationships which may be considered as potential competing interests: No conflict of interest other than MAG serving the IJCCHD editorial board but was not involved in the handling of the paper. If there are other authors, they declare that they have no known competing financial interests or personal relationships that could have appeared to influence the work reported in this paper.
